# Frame-based stereotactic biopsies of brainstem lesions – Monocentric comparison of the transfrontal and the suboccipital-transcerebellar approach over a 16-year period

**DOI:** 10.1007/s10143-024-03075-8

**Published:** 2024-11-01

**Authors:** Manuel Kaes, Jan-Oliver Neumann, Christopher Beynon, Paul V. Naser, Karl Kiening, Sandro M. Krieg, Martin Jakobs

**Affiliations:** 1 Department of Neurosurgery, Heidelberg University Hospital, Im Neuenheimer Feld 400, 69120 Heidelberg, Germany; 2 Division for Stereotactic Neurosurgery, Department of Neurosurgery, Heidelberg University Hospital, Im Neuenheimer Feld 400, 69120 Heidelberg, Germany; 3 Medical Faculty, Heidelberg University, Grabengasse 1, 69117 Heidelberg, Germany

**Keywords:** Stereotactic biopsy, Brainstem, Transfrontal approach, Suboccipital-transcerebellar approach

## Abstract

Both the transfrontal and the suboccipital-transcerebellar approach are frequently used trajectories for frame-based stereotactic biopsies of brainstem lesions. Nevertheless, it remains unclear which approach is more favorable in terms of complications, diagnostic success and outcome, especially considering the location of the lesion within the brainstem. This study compared the safety and diagnostic yield of these two approaches. Furthermore, a brainstem zone model was created to answer the question, whether there is a favorable approach depending on the location of the lesion in the brainstem. A retrospective analysis of 84 consecutive cases of frame-based stereotactic biopsies for brainstem lesions via either transfrontal or suboccipital-transcerebellar approaches over a 16-year period was performed. Clinical and surgical data regarding trajectories, histopathology, complications and outcome was collected. The brainstem was divided in anatomical zones to compare the use of the two approaches depending on the location of the lesions. A total of *n* = 84 cases of stereotactic biopsies for brainstem lesions were performed. In 36 cases the suboccipital-transcerebellar approach was used, while in 48 cases surgery was performed via the transfrontal approach. The patient’s demographic data were comparable between the two approaches. Overall diagnostic yield was 90.5% (93.8% transfrontal vs. 86.1% suboccipital, *p* = 0.21, Risk Difference (RD) 0.077, CI [-0.0550, 0.2090]). Complications occurred in 11 cases (total complication rate: 13.1%; 12.5% transfrontal vs. 13.9% suboccipital, *p* = 0.55, RD 0.014, CI [-0.1607, 0.1327]). The brainstem model showed a more frequent use of the suboccipital approach in lesions of the dorsal pons. The transfrontal approach was used more frequently in mesencephalic targets. No significant differences in terms of complications and diagnostic yield were observed, even though complications in medullary lesions appeared higher using the transfrontal approach. This study showed, that if the approaches are used for their intended target locations there are no significant differences between the transfrontal and the suboccipital-transcerebellar approach for frame-based stereotactic biopsies of brainstem lesions in terms of diagnostic yield and safety. Therefore, our data suggests that both approaches should be considered for stereotactic biopsy of brainstem lesions.

## Introduction

Frame-based stereotactic biopsies of lesions within the brainstem are important for diagnosis and therapeutic modalities in non-resectable lesions. Recent studies showed a clinically relevant discrepancy between the suspected radiological diagnosis and the final histopathological diagnosis after tissue asservation [14, 18, 20, 24]. Further, the benefits of a complete molecular pathological analysis with the opportunity for optimized, targeted therapy can only be embraced if tissue is acquired. Due to the high density of eloquent, function-bearing areas in the brainstem, frame-based stereotactic biopsy is known to be the safest and most reliable procedure to establish a diagnosis while preserving the neurological functions of the patients. Nevertheless, complications and mortality in frame-based stereotactic biopsies are more common when used in brainstem lesions compared to other areas of the brain [16, 26].

There are two commonly used approaches to reach brainstem lesions. First, the transfrontal approach, which requires a frontal burrhole and reaches the brainstem via the cerebrum. Second, the suboccipital-transcerebellar approach, which uses an occipital burrhole and reaches the brainstem through the cerebellum, more specific the cerebellar pedunculi. Both approaches have widely been used over many decades and the discussion, which approach is preferable, is still ongoing. Both approaches share a relatively high diagnostic yield and low rate of complication [11]. The benefit of the transfrontal approach is the convenient use with most stereotactic frames and the relatively low amount of frame adjustments and mounting changes offering a similar operative setting to biopsies in supratentorial areas, which most stereotactic neurosurgeons are familiar with. On the downside the lengths of the trajectories through the healthy brain tissue are longer and CSF-spaces as well as vasculature may require trajectory adjustments. The suboccipital approach offers a short trajectory through healthy brain tissue but usually traverses more eloquent areas and is technically more demanding. There are few publications comparing both approaches in terms of complications and diagnostic yield, predominantly with only limited case numbers [7, 11]. The question, which approach is preferable when considering the location of the brainstem lesion, remains unclear and is mostly a case-based decision influenced by the preference of the attending surgeon.

At our institution both approaches have been widely used for the past two decades. In this study we focused on the complications and diagnostic yield of both approaches, correlated them with the location of the lesion within the brainstem and generated a brainstem-zone model to assess the question, whether there are specific regions within the brainstem where one of the two approaches is preferable.

## Methods

### Study design

For this retrospective study all frame-based stereotactic surgeries at our hospital from 2007 to March 2023 were screened. All cases with biopsies harboring the target point within the brainstem (mesencephalon, pons, medulla) were included and divided in two groups depending on the approach used. Most surgeries were performed by 1 out of 3 board certified neurosurgeons with stereotactic specialization.

### Surgical technique

All patients were operated under general anesthesia and with intraoperative stereotactic imaging. After orotracheal intubation and initiation of general anesthesia, the patient´s head was fixed in the stereotactic frame (titanium frame, inomed Medizintechnik GmbH, Emmendingen, Germany; legacy carbon frame, Fischer-Leibinger, Stetten, Germany). While the supratentorial approach requires the frame to be mounted low (positive orientation) with long mounting posts, the suboccipital approach requires the frame to be mounted high, in an upside down (negative orientation) fashion with short mounting posts, high enough to allow the blades of the stereotactic localizer to be mounted without colliding with the patient´s shoulders. The frame is mounted with a predefined pitch, parallel to the canthomeatal line with the head centered to eliminate roll as well as yaw and fixation pins are placed in the orbital rim and the occipital area. Localizer blades are added to the stereotactic frame and the patient is placed in the intraoperative CT or MRI scanner. Images are acquired in supine position with both techniques. For intraoperative CT scans, contrast-enhanced 1 mm axial slices with isotropic voxels were acquired. For intraoperative MRI scans, a 1 mm contrast enhanced T1 sequence (T1-VIBE 3D) was acquired as the stereotactic image series. In cases of non-contrast enhancing lesions a 2 mm sliced FLAIR sequence was added to visualize the target lesion. The localizer blades are removed from the ring and the patient is placed on the operating table in the supine position for the transfrontal approach. For the suboccipital approach the patient’s head is turned to the contralateral side of the planned entry point and the neck is slightly inclined to expose the suboccipital area. The ipsilateral shoulder is slightly elevated and bolstered, if necessary, taped down to increase working space. The stereotactic frame is then attached to the operating table via a Mayfield-type adapter. The planning of the trajectory is performed using the stereotactic planning software (Inomed Planning System iPS V4.0–7.0, inomed Medizintechnik GmbH, Emmendingen, Germany). After automated image fusion a safe trajectory, which avoids sulci, ventricles and vessels is planned. The entry point is then shaved, properly disinfected and draped in a sterile fashion. The stereotactic system is then mounted. For the suboccipital approach the Zamorano-Duchovny (ZD) frame (Inomed Medizintechnik GmbH, Emmendingen, Germany) was used, for the transfrontal approach also the Riechert-Mundinger frame (Inomed Medizintechnik GmbH, Emmendingen, Germany) was used for its three-point fixation and stable nature for the longer transfrontal trajectories. After linear skin incision at the entry point, bipolar coagulation of bleeding and placement of a small retractor a 12 mm burrhole is made and the revealed dura is opened with a blade using the cruciate technique. The cortex is avascularized. The biopsy cannula is stereotactically placed with the instrument holder of the stereotactic system set to the depth of the first biopsy specimen to be taken. The burrhole is then sealed with a gelatine sponge and fibrin glue to minimize loss of cerebrospinal fluid and therefore potential brain shift. Specimens are then taken in a serial fashion using a stereotactic biopsy forceps (Inomed Medizintechnik GmbH) that is advanced in 1 mm steps until the last desired specimen is taken at the target point level. Specimens are then transferred individually into formalin and sent to the neuropathological department for analysis. After the last specimen has been taken, the biopsy cannula is removed and a gelatine sponge seal is placed inside the burrhole. Wound closure is then performed in a layered fashion before the wound gets disinfected and draped. The stereotactic frame is then removed before general anesthesia is terminated and the patient extubated.

### 3D Model

**Fig. 1:**
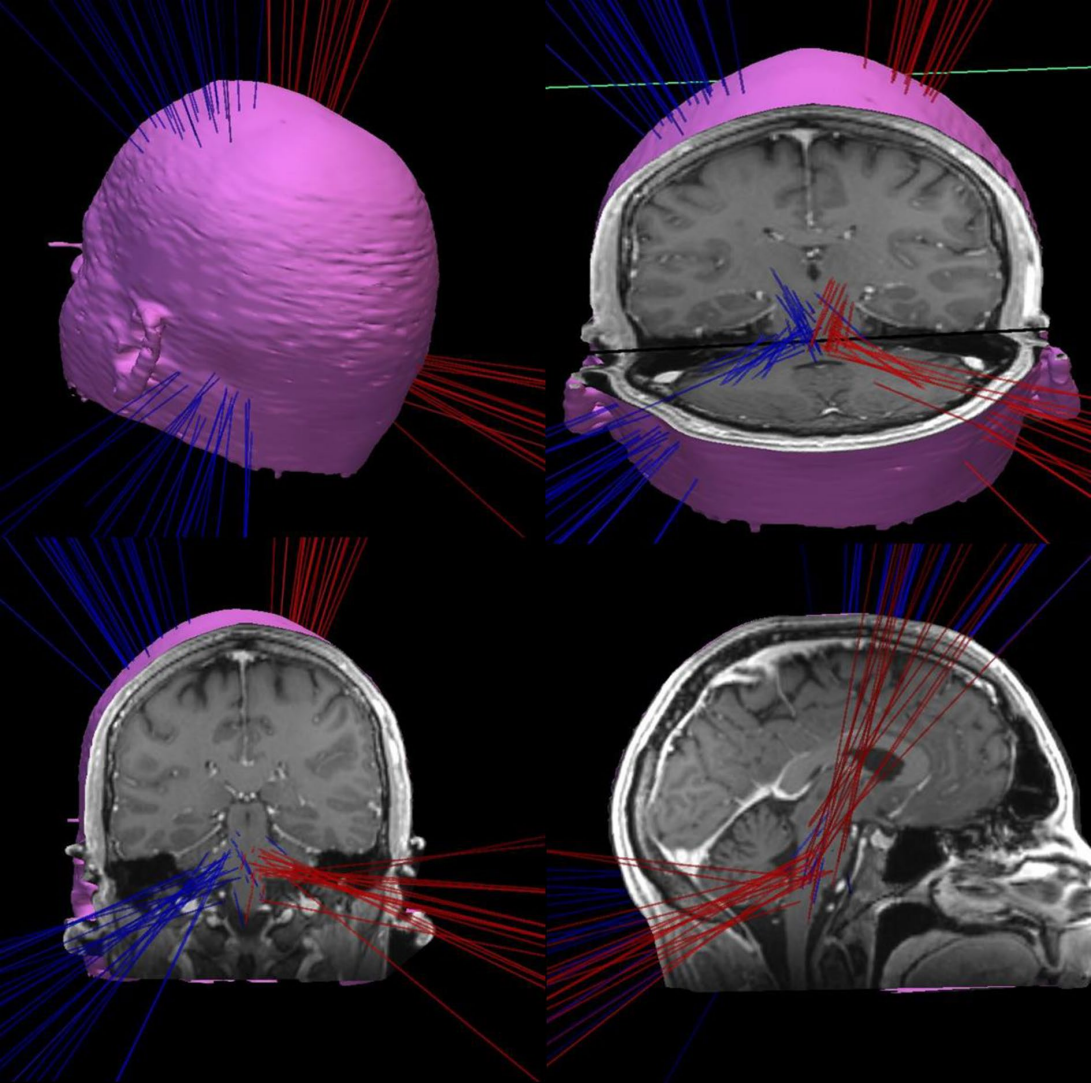
3D-model of the analyzed trajectories projected in a T1-MRI. Red = trajectories with entry point on the right side, blue = trajectories with entry point on the left side

The 3D Model for visualizing of the trajectories was created using the Inomed planning software (Inomed Medizintechnik GmbH, Emmendingen, Germany). Coordinates of target- and entrypoints for each trajectory were determined according to the ac-pc constant. Then, trajectories were inserted in the T1 MRI data set of a healthy, adult male individual under the four eyes principle (Fig. 1).

### Clinical data

Assessment of clinical data was done using the hospital records regarding progress reports, discharge letters, surgical letters, admission letters and lab results. Histopathological data was taken from the reports of the department of neuropathology, Heidelberg University Hospital, Heidelberg, Germany and classified as a “diagnostic success”, if a proper neuropathological diagnosis was achieved or “non-diagnostic”, if no proper diagnosis could be established. Analysis of pre-, intra- and postoperative MRI and CT-scans was done with the Picture Archiving and Communication System (PACS). ASA-score was assessed using the most recent examination documentation prior to surgery. Any change in the neurological state of a patient after surgery was considered as a new neurological deterioration and additionally classified as “temporary”, if completely resolved at discharge or “permanent” if still present at discharge. Parts of the clinical data was taken out of our stereotactic database and had been published previously [2, 3, 19].

### Statistics

Data are shown as mean (± standard deviation) or frequencies (percentage). Categorial variables were tested on significance using the chi-square test and fisher´s exact test, continuous variables with students t-test, respectively. All statistic calculations were made with SPSS Statistics (IBM, Version 29.0,2022). Significance level was defined as *p* < 0.05.

### Ethics

This study was approved by the ethic commission of the Heidelberg medical faculty, Heidelberg, Germany and conducted in accordance with the Declaration of Helsinki.

## Results

### Patient’s characteristics

**Fig. 2:**
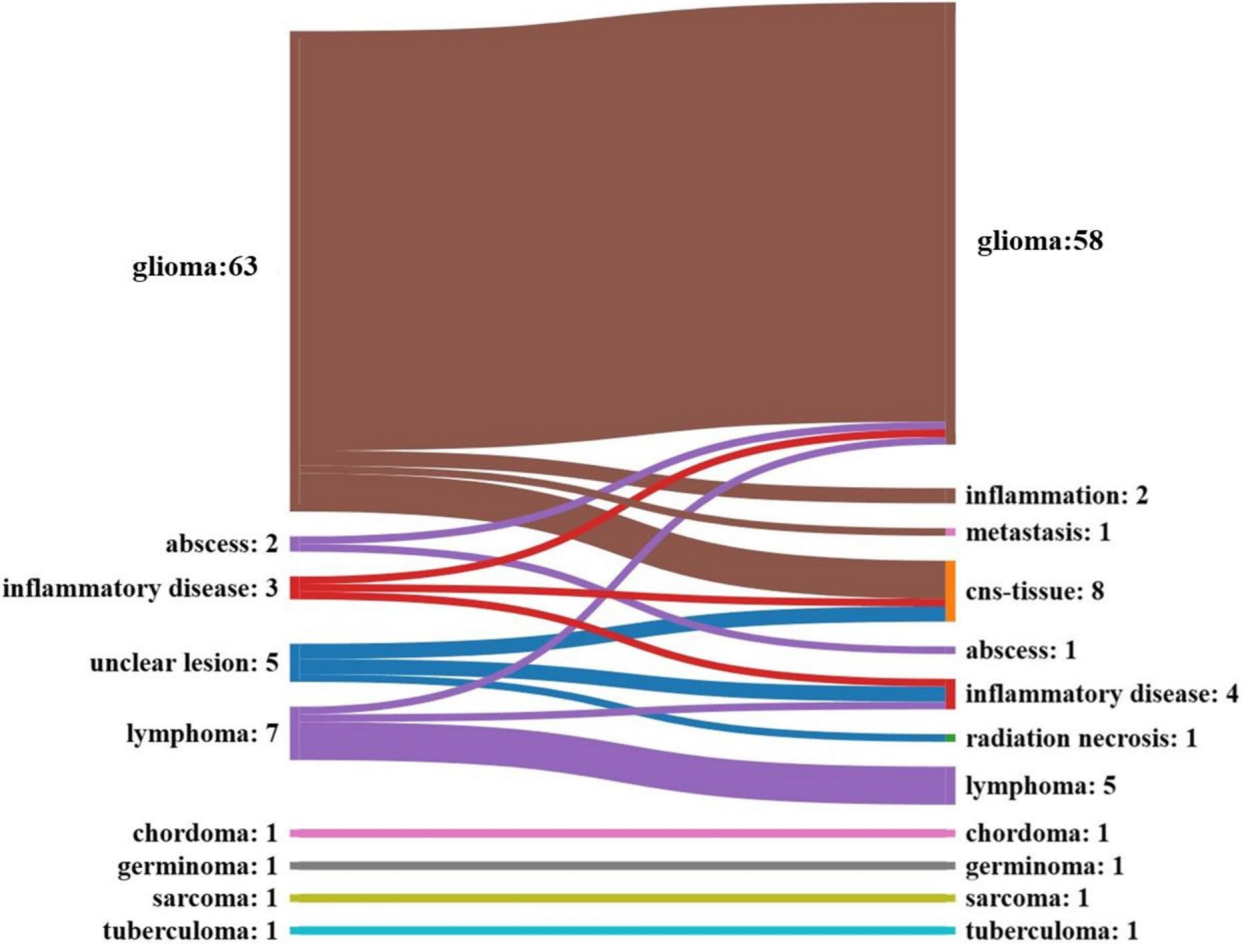
Flowchart of the suspected entity prior to surgery (left) and the final entity after histopathological analysis (right). Note, that the entity “cns-tissue” was considered non-diagnostic

From 2007 to 03.2023 a total of *n* = 84 stereotactic biopsies for brainstem lesions were performed. In 48 cases (57.1%) the transfrontal approach was used while in 36 cases (42.9%) the suboccipital approach was used. Mean age was 42.0 years (SD 21.3), and 43 patients (51.2%) were female. Descriptive parameters, ASA-Scores as well as histopathological diagnoses are shown in Table 1 and Fig. 2. There were no significant differences in patient characteristics when comparing the transfrontal and suboccipital case groups.

**Table 1:** Baseline characteristics with epidemiological parameters, American Society of Anesthesiologist (ASA) score, use of intraoperative CT (computed tomography scan) and MRI (magnetic resonance imaging), number of specimen and CT within one week after surgery, grouped for all patients and the two approaches, *p*-value

Parameter	Overall (%)	Transfrontal (%)	Suboccipital (%)	*P*-value
Case number	84	48 (57.1%)	36 (42.9%)	
Gender	41 m (*48.8%)*	27 (*56.3%)*	14 (*38.9%)*	0.09
	43f (*51.2%)*	21 (*43.8%)*	22 (*61.1%)*	
Age	2–82, mean 42 (+ -21.3)	2–82, mean 43.6 (+ -20.4)	5–79, mean 39.9 (+ -22.5)	0.22
ASA-score				0.108
1	40 (*47.6%)*	24 (*50%)*	16 (*44.4%)*	
2	28 (*33.3%)*	18 (*37.5%)*	10 (*27.8%)*	
3	13 (*15.5%)*	6 (*12.5%)*	7 (*19.4%)*	
4	3 (*3.6%)*	0	3 (*8.3%)*	
5	0	0	0	
CT	11 (*13.1%)*	8 (*16.7%)*	3 (*8.3%)*	0.22
MRI	73 (*86.9%)*	40 (*83.3%)*	33 (*91.7%)*	
Specimen	8–31, mean 14.8 (+ -4.2)	6–26, mean 14.5 (+ -4.1)	6–31, mean 15.3 (+ -4.5)	0.2
CT within 7 days	36 (*42.9%)*	24 (*50%)*	12 (*33.3%)*	0.96

### Diagnoses and histopathology

Most common diagnosis was glioma (58 cases, 69%) followed by inflammatory disease (6 cases, 7.1%) and lymphoma (5 cases, 6%). Other diagnoses were abscess, germinoma, metastasis, sarcoma, tuberculoma, chordoma and radiation necrosis (1 case, 1.2% each). In 8 cases (9.5%) a definitive diagnosis could not be established. These cases were classified as non-diagnostic (see also Fig. 2). The brain tumors were further classified according to the current WHO classification. The grading showed WHO 1 in 7 cases (12.3%), WHO 2 in 9 cases (15.8%), WHO 3 in 16 cases (28.1%) and WHO 4 in 25 cases (43.9%) while 1 case could not be assigned to a certain WHO grade. We found a match of the radiological estimated diagnoses with the histopathological diagnoses regarding the entity of the lesions in 66 cases (78.6%).

### Surgical procedure

Most of the surgeries were performed using an intraoperative MRI (73 cases, 86.9%), while 11 cases (13.1%) were performed with an intraoperative CT-Scan for acquisition of stereotactic images. The number of acquired specimen ranged between 8 and 31 (mean 14.8, SD 4.2). A postsurgical CT scan within 1 week following the procedure was performed in 36 cases (42.9%). We found no significant differences between the two approaches regarding any surgical parameter (details in Table 1).

### Brainstem model

**Fig. 3:**
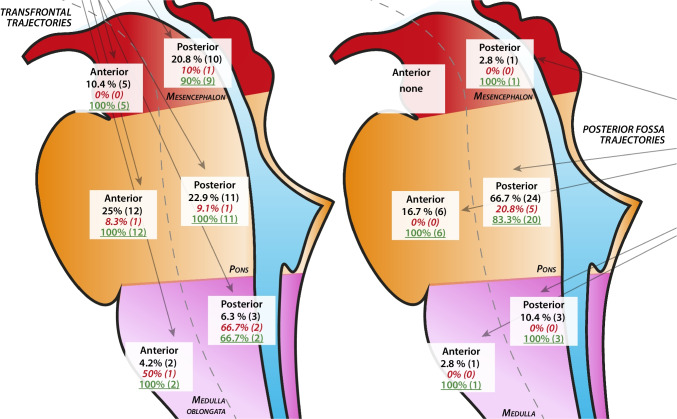
Brainstem zone model for the transfrontal trajectories and the suboccipital trajectories with division of the brainstem in the following parts: mesencephalon, ventral and dorsal, pons, ventral and dorsal and medulla, ventral and dorsal. Shown are the total number of biopsies performed (black, first line, Percent of the total number of biopsies performed with each approach), the number of complications (red, italics, second line, percent of the total number of performed biopsies for each brainstem zone) and the diagnostic yield (green, third line, percentage of the total number of biopsies performed for each brainstem zone)

**Fig. 4:**
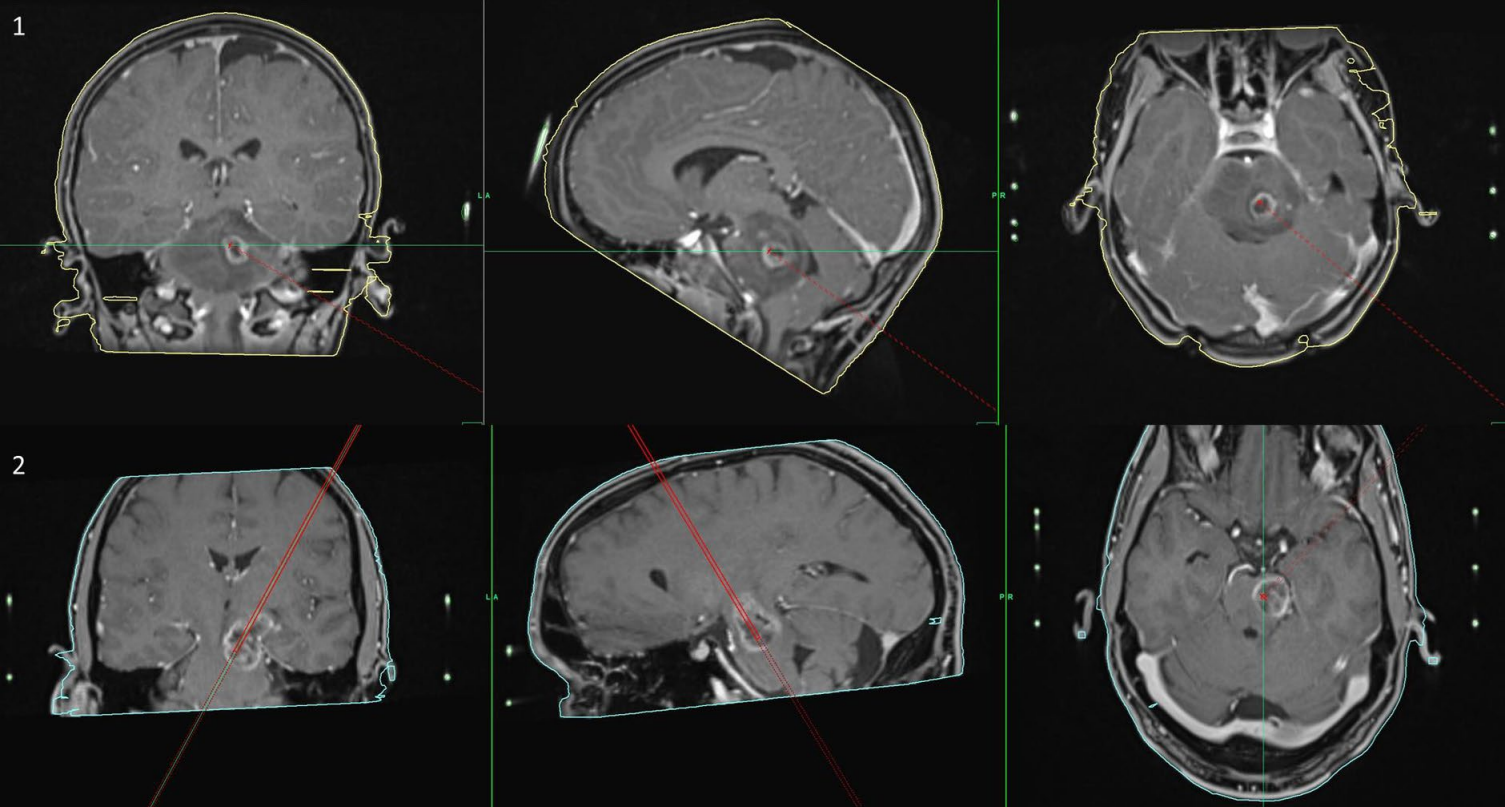
Two illustrative cases with the used trajectories for biopsy. 1: shown is an unclear lesion in the pons, biopsy was performed via the suboccipital approach, histopathological analysis revealed glioblastoma (T1-MRI with contrast sequence, coronar, sagittal and axial view). 2: shown is an unclear lesion in the mesencephalon, biopsy was performed via the transfrontal approach, histopathological analysis revealed a glioblastoma (T1-MRI with contrast sequence, trajectory long-view)

For creation of our brainstem model data of 78 patients were available (43 transfrontal, 35 suboccipital). The brainstem was divided in 6 zones: mesencephalon (ventral and dorsal), pons (ventral and dorsal) and medulla (ventral and dorsal). Most target points were located in the pons (18 ventral (23.4%) and 35 dorsal (45.5%)) followed by mesencephalon (5 ventral (6.5%) and 11 dorsal (14.3%)) and medulla (3 ventral (3.9%) and 6 dorsal (7.8%)). See also Tables 2, 3, 4 and Figs. 3 and 4.

**Table 2:** Diagnostic yield, complications, focal-neurological deficits (FND) and outcome parameters for all patients and grouped for the used approaches, p-values, Risk Difference (RD) and 95% confidence interval (CI)

Parameter	Overall *n* = 84 (%)	Transfrontal *n* = 48 (%)	Suboccipital *n* = 36 (%)	P-value	RD	CI
Diagnostic yield	76 (*90.5%)*	45 (*93.8%)*	31 (*86.1%)*	0.21	0.077	[-0.0550, 0.2090]
Complications	11 (*13.1%)*	6 (*12.5%)*	5 (*13.9%)*	0.55	0.014	[-0.1607, 0.1327]
FND permanent	6 (*7.1%)*	4 (*8.3%)*	2 (*5.6%)*	0.48	0.027	[-0.0813, 0.1353]
FND transient	5 (*6%)*	2 (*4.2%)*	3 (*8.3%)*	0.37	0.041	[-0.1475, 0.0655]
Hemorrhage	7 (*8.3%)*	3 (*6.3%)*	4 (*11.1%)*	0.34	0.048	[-0.1715, 0.0755]
30-day mortality	4 (*4.8%)*	1 (*2.1%)*	3 (*8.3%)*	0.21	0.062	[-0.1608, 0.0368]

**Table 3:** Brainstem zone model with the number of target points, transfrontal approach, suboccipital approach, complications and diagnostic yield for each brainstem zone

Brainstem area	Target- points n (%)	Transfrontal *n* (%)	Suboccipital *n* (%)	Complications *n* (%)	Diagnostic yield *n* (%)
Mesencephalon, ventral	5 *(6.5%)*	5 *(100%)*	0	0	5 *(100%)*
Mesencephalon, dorsal	11 *(14.3%)*	10 *(91%)*	1 *(9.1%)*	1 *(9.1%)*	10 *(90.9%)*
Pons, ventral	18 *(23.4%)*	12 *(66.7%)*	6 *(33.3%)*	1 *(5.6%)*	18 *(100%)*
Pons, dorsal	35 *(45.5%)*	11 *(31.4%)*	24 *(68.6%)*	6 *(17.1%)*	31*(88.6%)*
Medulla, ventral	3 *(3.9%)*	2 *(66.7%)*	1 *(33.3%)*	1 *(33.3%)*	3 *(100%)*
Medulla, dorsal	6 *(7.8%)*	3 *(50%)*	3 *(50%)*	2 *(33.3%)*	5 *(83.3%)*

**Table 4:** Brainstem zone model with the comparison of the transfrontal and the suboccipital trajectories regarding diagnostic yield and complications in each brain stem zone. RD = Risk Difference, CI = 95% confidence interval

Brainstem area	Complications Transfrontal	Complications Suboccipital	RD (CI)	Diagnostic Yield Transfrontal	Diagnostic Yield Suboccipital	RD (CI)
Mesencephalon, ventral	0%	-	N/A	100%	-	N/A
Mesencephalon, dorsal	10%	0%	0.1 (-0.0859, 0.2859)	90%	100%	0.1 (-0.2859, 0.0859)
Pons, ventral	8.3%	0%	0.083 (-0.0731, 0.2391)	100%	100%	0 (0, 0)
Pons, dorsal	9.1%	20.8%	0.117 (-0.1091, 0.2495)	100%	83.3%	0.167 (0.0178, 0.3162)
Medulla, ventral	50%	0%	0.5 (-0.1930, 1.1930)	100%	100%	0 (0, 0)
Medulla, dorsal	66.7%	0%	0.667 (0.1337, 1.2003)	66.7%	100%	0.333 (-0.8663, 0.2003)

### Mesencephalon

For biopsies in the Mesencephalon the transfrontal approach was almost exclusively used (ventral Mesencephalon: 5 out of 5 cases (100%), dorsal Mesencephalon: 10 out of 11 cases (91%)). The suboccipital approach was used in only 1 case (9%) in the dorsal mesencephalon. The more frequent use of the transfrontal approach proved to be statistically significant (*p* = 0.009). The overall diagnostic yield was 100% in the ventral mesencephalon and 90.9% in the dorsal mesencephalon. The transfrontal approach showed a diagnostic yield of 100% in the ventral mesencephalon and 90% in the dorsal mesencephalon while complications occurred in 0% ventral and in 10% dorsal. The only case with usage of the suboccipital approach showed no complications (0%) and a diagnosis could be established (100% diagnostic yield). The mesencephalon was traversed to reach lesions in the lower or more dorsally located brainstem areas in 33 cases (42.9%) all of which were performed via the transfrontal approach.

### Pons

For pontine lesions a more balanced use of the two trajectories was observed. The transfrontal approach was used in 12 out of 18 (66.7%) ventral pontine lesions and in 11 out of 35 (31.4%) dorsal pontine lesions. The complication rates for the transfrontal approach were 8.3% (*n* = 1) for ventral lesions and 9.1% (*n* = 1) for dorsal lesions while the diagnostic yield was 100% for both pontine areas. The suboccipital approach was used in 6 ventral cases (33.3%) and in 24 dorsal cases (68.6%). The diagnostic yield was 100% for ventral and 83.3% for dorsal lesions while complications occurred in 0% of the ventral and in 20.8% of the dorsal lesions. There was a significant difference regarding the use of the trajectories in the dorsal pons in favor of the suboccipital approach (*p* < 0.001). The approach usage in the ventral pons as well as the diagnostic yield and frequency of complications in both areas of the pons did not show any significant differences. Overall, the diagnostic yield in the ventral Pons was 100% and 88.6% in the dorsal pons, the total rate of complication was 5.6% in the ventral pons and 17.1% in the dorsal pons, respectively.

### Medulla

The medulla was the brainstem area least often biopsied; 3 biopsies were performed in the ventral part and 6 in the dorsal part. The total complication rate was 33.3% for both areas. The overall diagnostic yield was 100% ventrally and 83.8% dorsally. The transfrontal approach was used in 2 ventral cases and in 3 dorsal cases, respectively. Complications occurred in 1 case in the ventral and 2 cases in the dorsal medulla. Diagnostic yield showed a 100% success rate in ventral lesions and 66.7% in dorsal lesions. The suboccipital approach was used in 1 case for ventral and in 3 cases for dorsal biopsies. No complications occurred and a proper diagnosis could be achieved in every case. No significant differences regarding the use of the approaches, the diagnostic yield and the complication rate were found in the medulla, even though complications in the dorsal medulla appeared more frequent using the transfrontal approach. For more detailed information about the brainstem zones, diagnostic yield and complications see Table 3 and Fig. 3.

### Diagnostic yield

The overall diagnostic yield was 90.5% (76 of 84 cases). The transfrontal approach showed a diagnostic yield of 93.8% (45 of 48 cases) compared to 86.1% (31 of 36 cases) for the suboccipital approach. The difference appeared to be statistically insignificant (*p* = 0.21, RD 0.077, CI [-0.0550, 0.2090]). The number of specimen was comparable between the two approaches (14.5 transfrontal, 15.3 suboccipital, *p* = 0.2). Follow-up of the non-diagnostic cases showed 3 patients which underwent another stereotactic biopsy with successful proof of a tumor, 1 patient had progression of multifocal lesions which lead to open surgery and an abscess was diagnosed, 1 patient was lost in the follow up, 3 patients showed no clinical or radiological progress during follow up with the suspicion of a tumor being successfully ruled out.

Post-surgical image analysis allowed reconstruction of trajectories which showed that the biopsies had been taken from inside the desired lesions. Surgical accuracy therefore was 100% in the non-diagnostic cases.

### Complications

Overall, complications occurred in 11 cases (13.1%). These consisted of new neurological deficits in all cases, which were permanent in more than half of the cases (6 cases (7.1%)). Hemorrhage was detected in 7 cases (8.3%). All cases with hemorrhage showed new neurological deterioration (5 cases (6.0%) permanent, 2 cases (2.4%) transient). Total complication rate for the transfrontal approach was 12.5% (6 cases) with permanent deficits in 4 cases (8.3%) and transient deficits in 2 cases (4.2%). After CT-scan we found additional hemorrhage in 3 cases (6.3%) all in the patients with persisting deficits. For the suboccipital approach complications were found in 5 cases (13.9%) with permanent deficits in 2 cases (5.6%), transient deficits in 3 cases (8.3%) and additional hemorrhage in 4 cases (11.1%, 2 cases with persisting deficits, and 2 cases with transient deficits). The differences between the two approaches appeared to be statistically insignificant (*p* = 0.55, RD 0.014, CI [-0.1607, 0.1327]).

### Outcome

To further determine the outcome of our patients collective the mortality rate within 30 days after surgery was assessed. Furthermore, 4 patients (4.8%) died within 30 days post-surgery caused by surgery related hemorrhage in 2 cases, non-surgery related disease progress with meningeosis gliomatosa in 1 case and sepsis caused by immunodeficiency due to lymphoma in 1 case. For the transfrontal group this occurred in 1 case (2.1%) and for the suboccipital group in 3 cases (8.3%). Median ASA-Score was 3 in the early mortality group, compared to 2 in the other patients, a difference which was statistically significant (*p* < 0.02).

## Discussion

### Frequency of the used approaches

Based on our experience, the primary considerations for determining the most appropriate approach include the ability to plan a safe trajectory that reaches the target point without encountering vascular conflicts. Additionally, the trajectory should warrant a maximal safe number of specimen. Lastly, the Trajectory should avoid ventricles as well as CSF spaces especially in elderly patients with ventiruclomegaly. The transfrontal approach may be challenging in these cases, as the ventricles cannot be avoided, which in turn could increase the risk of hemorrhage and trajectory deviation due to brain shift. Our data showed, that both approaches are safe and reliable. A more frequent use of the transfrontal approach was found in the mesencephalon as well as in the ventral parts of pons and medulla while the suboccipital approach was used more frequently in the dorsal parts of the pons. However, the differences in use of both approaches were only significant in the dorsal pons and the mesencephalon overall. In the published literature only one study regarding the use of both approaches in dependence of the location of the lesion in the brainstem was identified. Jaradat et al. (2021) reported similar findings with a higher frequency of use for the transfrontal trajectory for mesencephalic lesions and for the suboccipital approach for pontine lesions in 23 patients, respectively [13]. Our data supports and validates the findings of Jaradat et al. (2021) in a large cohort and the intuitive assumptions of some other published studies, that the suboccipital approach is more frequently used in the dorsal parts of the Pons. This may be attributed to the fact that the trajectory is shorter and the route alongside the cerebellar peduncles is safer [7, 9, 13].

### Diagnostic yield

Even though a higher diagnostic yield for the transfrontal approach (93.8% vs. 86.1%) was found, this difference was not statistically significant. While comparing the yield between the two approaches regarding the brainstem area we also found no significant differences. Our findings are in accordance with previous published literature [11, 13, 23]. For example Dellaretti et al. (2012) reported a diagnostic yield of 95.1% for the transfrontal approach and 84.2% for the suboccipital approach, also without significant differences in statistical analysis [7]. In general the diagnostic yield in brainstem lesions ranges from 86 to 100% [5, 8–10, 12, 13, 16, 17, 23]. This falls in line with our diagnostic yield of 90.5%. Therefore, we propose, that the diagnostic yield is not significantly affected by the approach used. When considering the suspected diagnosis prior to surgery compared to the final histopathological diagnosis, a high discrepancy of 21.4% was found. Therefore, we can add further evidence regarding the importance of biopsies in brainstem lesions and the additional value in diagnostics. This is in good accordance to previous published literature and a further supporting argument to perform biopsies for unclear brainstem lesions [14, 18, 20, 24].

### Complications

The complication rate between the two approaches showed a comparable occurrence of complications for both approaches (12.5% transfrontal vs 13.9% suboccipital). While differentiating the neurological deterioration after biopsy we found mostly cranial nerve palsies (CN VI and VII), limb paresis, limb hypesthesia, vertigo, dysarthria and decreased consciousness. In 7 cases, additionally hemorrhage was found with no significant differences between the two approaches. In general our complication rate is comparable to other published studies in which the complication rates for both approaches are up to 20% [5, 6, 8–10, 12, 16, 17, 21, 23, 26]. However, there are a few papers with a lower rate of complications up to 0% [1, 13, 18, 22]. These are mostly studies with a relatively small case number and different definitions of complications amongst the studies. In our study, complications were generously enclosed, even if only transient neurological deficits occurred, which totally receded at discharge. Further, like previously reported, the complication rate in pediatric patients appears lower than in adult patients [11]. Our cohort contained 12 pediatric patients (age under 18), which showed complications in 1 case (8.3%). Other published studies comparing both approaches did also find no significant differences regarding the complication rate between the two approaches in pediatric cases [10, 13]. Delarretti et al. (2012) also found no significant differences regarding frame-based stereotactic biopsies for brainstem lesions with a morbidity rate of 5.3% for 19 suboccipital approaches and 9.8% for 112 transfrontal approaches, which is still the study with the largest number of cases that compares both approaches [7]. While considering the brainstem areas in terms of complications we did not find any significant differences between the two approaches even though there was a fair trend (*p* = 0.08) towards a lower complication rate for the suboccipital approach in the dorsal medulla. Our brainstem model visualized an upwards trend for complication rates for the transfrontal approach the more caudally the biopsy was performed within the brainstem. This could be seen as evidence towards the hypothesis, that complications occur more frequently as the length of the trajectory through healthy brain tissue increases. To entirely elucidate the occurrence of complications regarding the length of trajectories, further studies with more patients are necessary. No publications comparing the safety of both approaches depending on the brainstem area were found in the available literature. To avoid hemorrhage after stereotactic biopsies further studies with respect to preoperative coagulopathy as well as risk factor assessment are necessary, as previously reported [2, 4].

### Outcome

In our cohort, 4 patients (4.8%) died within 30 days after surgery. The death of 2 patients in the 30-day mortality group showed a direct link to the surgery because of hemorrhage. One patient died within 30 days due to disease progression with leptomeningeal spread and hydrocephalus. Another patient died within 30 days due to sepsis associated with lymphoma and immunodeficiency, not related to surgery. We also found a significantly higher ASA-Score in the early mortality group (3 vs. 2, *p* < 0.02), giving further evidence the 30-day mortality is more dependent on the medical condition of the patient. Previous published studies reported a surgery related mortality rate of 0% to 5.3% [6, 7, 9, 16, 20, 23, 25]. A significant difference between the two approaches is not described [7, 23].

### Future aspects

For a definitive answer, which approach is preferable for frame-based stereotactic biopsies of brainstem lesions further studies and experiences from other centers are needed due to the relatively low prevalence of brainstem lesions. Further research should also address the high incongruity of estimated diagnosis and histopathological diagnosis to assess the entities, which can be diagnosed and treated with high accuracy without a biopsy to optimize the case selection and therefore avoid unnecessary biopsies. For example liquid biopsies—a technique, in which DNA is extracted from cerebrospinal fluid and analyzed—could add additional diagnostic value. However, further research is currently needed to determine, which entities can be reliably diagnosed with this still-emerging technique [27]. To maximize the diagnostic yield and minimize the complication rates the use of modern techniques, like artificial intelligence for trajectory planning or the use of Robot-assisted methods during the surgery could be a helpful tool in the future [15, 28].

### Summary

In this paper the question was assessed, if one of the most commonly used approaches for frame-based stereotactic biopsies of brainstem lesions is more favorable in terms of diagnostic yield and complications considering the location of the lesion in the brainstem. Overall, both approaches were found to be comparable safe and reliable. Our cohort, which represents the largest cohort when considering this question, showed no significant differences between the complication rate and diagnostic yield, instead demonstrating a trend towards a lower complication rate when using the suboccipital approach for lesions in the medulla. In our hospital, the transfrontal approach was used more frequently for lesions in the mesencephalon and the ventral parts of the pons while the suboccipital approach was more frequently used in the dorsal pons. Therefore, under the precondition of a similar localization dependent use, both approaches should be considered while planning a trajectory to reach brainstem lesions and should be a part of the repertoire of stereotactic neurosurgeons.

## Data Availability

The datasets generated and analyzed during the current study are available from the corresponding author on reasonable request.

## References

[1] Bahrami E, Parvaresh M, Bahrami M, Fattahi A (2020) An Experience with Frame-Based Stereotactic Biopsy of Posterior Fossa Lesions via Transcerebellar Route. World Neurosurg 136:e380–e385. 10.1016/j.wneu.2020.01.003 31931238 10.1016/j.wneu.2020.01.003

[2] Beynon C, Hoffmann T, Wick W, Unterberg A, Kiening K (2011) Stereotactic brainstem biopsy in a patient with coagulopathy of unclear etiology: case report. Min - Minim Invasive Neurosurg 54:268–270. 10.1055/s-0031-1297989 22278794 10.1055/s-0031-1297989

[3] Beynon C, Neumann J-O, Bösel J, Unterberg AW, Kiening KL (2013) Stereotactic biopsy and drainage of a brainstem abscess caused by listeria monocytogenes: —case report—. Neurol Med Chir (Tokyo) 53:263–265. 10.2176/nmc.53.263 23615421 10.2176/nmc.53.263

[4] Beynon C, Wei S, Radbruch A, Capper D, Unterberg AW, Kiening KL (2018) Preoperative assessment of haemostasis in patients undergoing stereotactic brain biopsy. J Clin Neurosci 53:112–116. 10.1016/j.jocn.2018.04.035 29685415 10.1016/j.jocn.2018.04.035

[5] Chen S-Y, Chen C-H, Sun M-H, Lee H-T, Shen C-C (2011) Stereotactic biopsy for brainstem lesion: Comparison of approaches and reports of 10 cases. J Chin Med Assoc 74:110–114. 10.1016/j.jcma.2011.01.024 21421204 10.1016/j.jcma.2011.01.024

[6] Cheng G, Yu X, Zhao H, Cao W, Li H, Li Q, Li Z, Yin F, Liu R, Zhang J (2020) Complications of stereotactic biopsy of lesions in the sellar region, pineal gland, and brainstem: A retrospective, single-center study. Medicine (Baltimore) 99:e18572. 10.1097/MD.0000000000018572 32080071 10.1097/MD.0000000000018572 PMC7034708

[7] Dellaretti M, Reyns N, Touzet G, Dubois F, Gusmão S, Pereira JLB, Blond S (2012) Stereotactic biopsy for brainstem tumors: comparison of transcerebellar with transfrontal approach. Stereotact Funct Neurosurg 90:79–83. 10.1159/000335502 22286495 10.1159/000335502

[8] Escobar-Vidarte OA, Griswold DP, Orozco-Mera J, Mier-Garcia JF, Peralta Pizza F (2022) A case series of stereotactic biopsy of brainstem lesions through the transfrontal approach. J Neurol Surg Rep 83:e123–e128. 10.1055/s-0042-1758696 36467870 10.1055/s-0042-1758696 PMC9708407

[9] Furtak J, Śledzińska P, Bebyn MG, Szylberg T, Krajewski S, Birski M, Harat M (2021) Infratentorial stereotactic biopsy of brainstem and cerebellar lesions. Brain Sci 11:1432. 10.3390/brainsci11111432 34827431 10.3390/brainsci11111432 PMC8615913

[10] Hamisch C, Kickingereder P, Fischer M, Simon T, Ruge MI (2017) Update on the diagnostic value and safety of stereotactic biopsy for pediatric brainstem tumors: a systematic review and meta-analysis of 735 cases. J Neurosurg Pediatr 20:261–268. 10.3171/2017.2.PEDS1665 28621573 10.3171/2017.2.PEDS1665

[11] He L, He D, Qi Y, Zhou J, Yuan C, Chang H, Wang Q, Li G, Shao Q (2021) Stereotactic biopsy for brainstem lesions: a meta-analysis with noncomparative binary data. Cancer Control 28:107327482110598. 10.1177/10732748211059858 10.1177/10732748211059858 PMC8670786 34875878

[12] Hirano Y, Shinya Y, Aono T, Hasegawa H, Kawashima M, Shin M, Takami H, Takayanagi S, Umekawa M, Ikemura M, Ushiku T, Taoka K, Tanaka S, Saito N (2022) The role of stereotactic frame-based biopsy for brainstem tumors in the era of molecular-based diagnosis and treatment decisions. Curr Oncol 29:4558–4565. 10.3390/curroncol29070360 35877220 10.3390/curroncol29070360 PMC9318548

[13] Jaradat A, Nowacki A, Fichtner J, Schlaeppi J-A, Pollo C (2021) Stereotactic biopsies of brainstem lesions: which approach? Acta Neurochir (Wien) 163:1957–1964. 10.1007/s00701-021-04733-2 33538882 10.1007/s00701-021-04733-2 PMC8195881

[14] Jung I, Chang KW, Park SH, Moon JH, Kim EH, Jung HH, Kang S, Chang JH, Chang JW, Chang WS (2021) Stereotactic biopsy for adult brainstem lesions: A surgical approach and its diagnostic value according to the 2016 World Health Organization Classification. Cancer Med 10:7514–7524. 10.1002/cam4.4272 34510820 10.1002/cam4.4272 PMC8559459

[15] Machetanz K, Grimm F, Wang S, Schuhmann MU, Tatagiba M, Gharabaghi A, Naros G (2022) Rediscovery of the transcerebellar approach: improving the risk-benefit ratio in robot-assisted brainstem biopsies. Neurosurg Focus 52:E12. 10.3171/2021.10.FOCUS21359 34973665 10.3171/2021.10.FOCUS21359

[16] Malaizé H, Laigle-Donadey F, Riche M, Marijon P, Mokhtari K, Bielle F, Tran S, Nichelli L, Beccaria K, Idbaih A, Hoang-Xuan K, Touat M, Carpentier A, Mathon B, the PSL BRAIN-BIOPSY STUDY GROUP (2022) Roles and outcomes of stereotactic biopsy for adult patients with brainstem lesion. J Neurooncol 160:159–170. 10.1007/s11060-022-04129-x 36083426 10.1007/s11060-022-04129-x

[17] Nakagawa JM, Trippel M, Doostkam S, Mader I, Coenen VA, Reinacher PC (2018) The stereotactic suboccipitaltranscerebellar approach to lesions of the brainstem and the cerebellum. Clin Neurol Neurosurg 166:10–15. 10.1016/j.clineuro.2018.01.015 29358106 10.1016/j.clineuro.2018.01.015

[18] Navarro-Olvera JL, Aguado-Carrillo G, Vintimilla-Sarmiento JD, Parra-Romero G, Guartazaca-Guerrero MS, Carrillo-Ruiz JD (2022) Concordancia y rendimiento diagnóstico de biopsias estereotáxicas para fosa posterior: técnica y experiencia en un hospital de referencia. Cir Cir 90:6417. 10.24875/CIRU.21000237 10.24875/CIRU.21000237 35944421

[19] Neumann J-O, Campos B, Younes B, Jakobs M, Jungk C, Beynon C, von Deimling A, Unterberg A, Kiening K (2018) Frame-based stereotactic biopsies using an intraoperative MR-scanner are as safe and effective as conventional stereotactic procedures. PLoS ONE 13:e0205772. 10.1371/journal.pone.0205772 30352066 10.1371/journal.pone.0205772 PMC6198960

[20] Patel P, Balamurugan M (2009) Transcerebellar stereotactic biopsy for brainstem lesions in children. J Pediatr Neurosci 4:17. 10.4103/1817-1745.49101 21887169 10.4103/1817-1745.49101 PMC3162830

[21] Peciu-Florianu I, Legrand V, Monfilliette-Djelad A, Maurage C-A, Vannod-Michel Q, Blond S, Touzet G, Reyns N (2022) Frameless robot-assisted stereotactic biopsies for lesions of the brainstem—a series of 103 consecutive biopsies. J Neurooncol 157:109–119. 10.1007/s11060-022-03952-6 35083580 10.1007/s11060-022-03952-6

[22] Phi JH, Chung H-T, Wang K-C, Ryu SK, Kim S-K (2013) Transcerebellar biopsy of diffuse pontine gliomas in children: a technical note. Childs Nerv Syst 29:489–493. 10.1007/s00381-012-1933-3 23053360 10.1007/s00381-012-1933-3

[23] Quick-Weller J, Lescher S, Bruder M, Dinc N, Behmanesh B, Seifert V, Weise L, Marquardt G (2016) Stereotactic biopsy of brainstem lesions: 21 years experiences of a single center. J Neurooncol 129:243–250. 10.1007/s11060-016-2166-1 27291894 10.1007/s11060-016-2166-1

[24] Rachinger W, Grau S, Holtmannspotter M, Herms J, Tonn J-C, Kreth FW (2009) Serial stereotactic biopsy of brainstem lesions in adults improves diagnostic accuracy compared with MRI only. J Neurol Neurosurg Psychiatry 80:1134–1139. 10.1136/jnnp.2009.174250 19520698 10.1136/jnnp.2009.174250

[25] Riche M, Amelot A, Peyre M, Capelle L, Carpentier A, Mathon B (2021) Complications after frame-based stereotactic brain biopsy: a systematic review. Neurosurg Rev 44:301–307. 10.1007/s10143-019-01234-w 31900737 10.1007/s10143-019-01234-w

[26] Riche M, Marijon P, Amelot A, Bielle F, Mokhtari K, Chambrun MPD, Joncour AL, Idbaih A, Touat M, Do C-H, Deme M, Pasqualotto R, Jacquens A, Degos V, Shotar E, Chougar L, Carpentier A, Mathon B (2022) Severity, timeline, and management of complications after stereotactic brain biopsy. J Neurosurg 136:867–876. 10.3171/2021.3.JNS21134 34507289 10.3171/2021.3.JNS21134

[27] Simonelli M, Dipasquale A, Orzan F, Lorenzi E, Persico P, Navarria P, Pessina F, Nibali MC, Bello L, Santoro A, Boccaccio C (2020) Cerebrospinal fluid tumor DNA for liquid biopsy in glioma patients’ management: Close to the clinic? Crit Rev Oncol Hematol 146:102879. 10.1016/j.critrevonc.2020.102879 32005411 10.1016/j.critrevonc.2020.102879

[28] Starup-Hansen J, Williams SC, Funnell JP, Hanrahan JG, Islam S, Al-Mohammad A, Hill CS (2023) Optimising trajectory planning for stereotactic brain tumour biopsy using artificial intelligence: a systematic review of the literature. Br J Neurosurg 1–10. 10.1080/02688697.2023.2210225 10.1080/02688697.2023.2210225 37177983

